# 4-Formyl-3-*p*-tolyl­sydnone

**DOI:** 10.1107/S1600536810016417

**Published:** 2010-05-08

**Authors:** Jia Hao Goh, Hoong-Kun Fun, Nithinchandra, B. Kalluraya

**Affiliations:** a X-ray Crystallography Unit, School of Physics, Universiti Sains Malaysia, 11800 USM, Penang, Malaysia; b Department of Studies in Chemistry, Mangalore University, Mangalagangotri, Mangalore 574 199, India

## Abstract

In the title compound, C_10_H_8_N_2_O_3_, the oxadiazole ring is essentially planar, with a maximum deviation of 0.006 (1) Å for the two-connected N atom. The mean planes through the aldehyde unit and the methyl-substituted phenyl ring make inter­planar angles of 13.60 (9) and 59.69 (4)°, respectively, with the oxadiazole ring. In the crystal structure, adjacent mol­ecules are inter­connected into a two-dimensional array parallel to (100) by inter­molecular C—H⋯O hydrogen bonds.

## Related literature

For general background to and applications of sydnone compounds, see: Hedge *et al.* (2008 ▶); Rai *et al.* (2008 ▶). For related sydnone structures, see: Baker & Ollis (1957 ▶); Grossie *et al.* (2009 ▶). For the stability of the temperature controller used for the data collection, see: Cosier & Glazer (1986 ▶). For bond-length data, see: Allen *et al.* (1987 ▶).
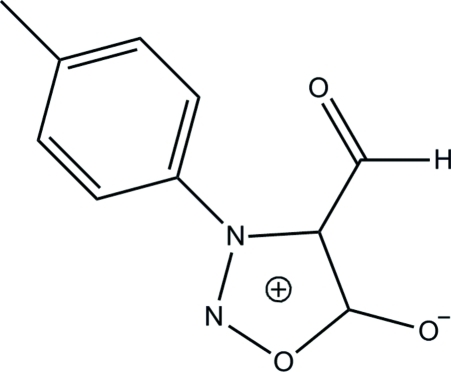


## Experimental

### 

#### Crystal data

C_10_H_8_N_2_O_3_
*M* *_r_* = 204.18
Monoclinic, 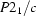

*a* = 10.5663 (4) Å
*b* = 10.4088 (3) Å
*c* = 8.9630 (3) Å
β = 108.222 (1)°
*V* = 936.34 (5) Å^3^
*Z* = 4
Mo *K*α radiation
μ = 0.11 mm^−1^
*T* = 100 K
0.71 × 0.30 × 0.19 mm

#### Data collection

Bruker APEXII DUO CCD area-detector diffractometer
Absorption correction: multi-scan (*SADABS*; Bruker, 2009 ▶) *T*_min_ = 0.926, *T*_max_ = 0.980
14437 measured reflections
4906 independent reflections
4091 reflections with *I* > 2σ(*I*)
*R*_int_ = 0.022

#### Refinement

*R*[*F* ^2^ > 2σ(*F* ^2^)] = 0.042
*wR*(*F* ^2^) = 0.132
*S* = 1.07
4906 reflections
137 parameters
H-atom parameters constrained
Δρ_max_ = 0.65 e Å^−3^
Δρ_min_ = −0.31 e Å^−3^

### 

Data collection: *APEX2* (Bruker, 2009 ▶); cell refinement: *SAINT* (Bruker, 2009 ▶); data reduction: *SAINT*; program(s) used to solve structure: *SHELXTL* (Sheldrick, 2008 ▶); program(s) used to refine structure: *SHELXTL*; molecular graphics: *SHELXTL*; software used to prepare material for publication: *SHELXTL* and *PLATON* (Spek, 2009 ▶).

## Supplementary Material

Crystal structure: contains datablocks global, I. DOI: 10.1107/S1600536810016417/fj2294sup1.cif

Structure factors: contains datablocks I. DOI: 10.1107/S1600536810016417/fj2294Isup2.hkl

Additional supplementary materials:  crystallographic information; 3D view; checkCIF report

## Figures and Tables

**Table 1:** Hydrogen-bond geometry (Å, °)

*D*—H⋯*A*	*D*—H	H⋯*A*	*D*⋯*A*	*D*—H⋯*A*
C1—H1*A*⋯O2^i^	0.93	2.41	3.2847 (9)	156
C5—H5*A*⋯O3^ii^	0.93	2.60	3.3489 (11)	138

Symmetry codes: (i) 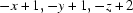; (ii) 
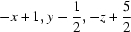
.

**Fig. 1.**
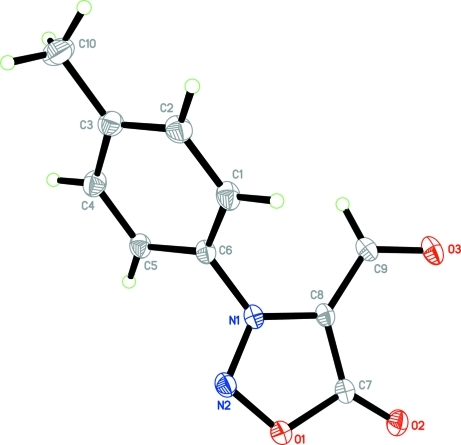
The molecular structure of the title compound, showing 50% probability displacement ellipsoids for non-H atoms and the atom-numbering scheme.

**Fig. 2.**
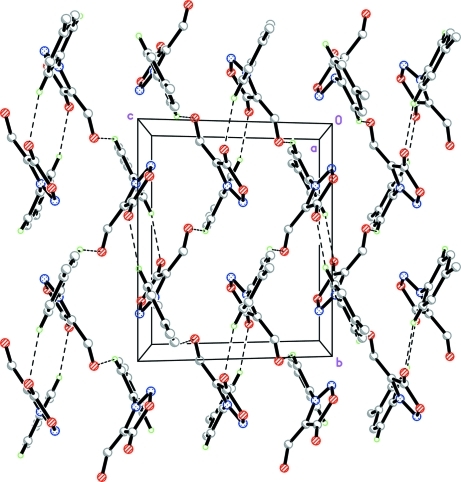
The crystal structure of the title compound, viewed along the a axis, showing a two-dimensional array parallel to the (100) plane. Hydrogen atoms not involved in intermolecular interactions (dashed lines) have been omitted for clarity.

## supplementary crystallographic information

### Comment

Sydnones constitute a well-defined class of mesoionic compounds consisting of 1,2,3-oxadiazole ring system. The introduction of the concept of mesoionic structure for certain heterocyclic compounds in the year 1949 has proved to be fruitful development in heterocyclic chemistry. The study of sydnones still remains a field of interest because of their electronic structure and also because of the various types of biological activities displayed by some of them. Interest in sydnone derivatives has also been encouraged by the discovery that they exhibit various pharmacological activities (Hedge *et al.*, 2008; Rai *et al.*, 2008).

These 4-formyl sydnone will be used for the preparation of a new series of α,β-unsaturated carbonyl compounds (namely chalcones) by condensation with appropriate ketones or aldehydes. These α,β-unsaturated carbonyl compounds will be utilized for the synthesis of a variety of novel heterocyclic compounds like pyrazolines, pyrazole *etc* carrying sydnone moiety.

Sydnones are mesoionic compounds containing a five-membered heterocyclic ring. Generally, substitution at the N3 position by an aromatic substituent is necessary for stability. In the title sydnone compound (Fig. 1), the aromatic substituent is *p*-toluene. The 1,2,3-oxadiazole ring (N1/N2/O1/C7/C8) in the sydnone unit is essentially planar, with maximum deviation of -0.006 (1) Å at atom N2. The mean planes through the aldehyde moiety (C9/H9A/O3) and methyl-substituted phenyl ring (C1-C6) are inclined at dihedral angles of 13.60 (9) and 59.69 (4)°, respectively, with the 1,2,3-oxadiazole ring. As reported previously (Grossie *et al.*, 2009), the exocyclic C7–O2 bond length of 1.2089 (9) Å is inconsistent to the formulation of Baker & Ollis (1957), which reported the delocalization of a positive charge in the ring, and a negative charge in the exocyclic oxygen. The bond lengths (Allen *et al.*, 1987) and angles are within normal range and comparable to a related sydnone structure (Grossie *et al.*, 2009). In the crystal structure (Fig. 2), intermolecular C1—H1A···O2 and C5—H5A···O3 hydrogen bonds (Table 1) link adjacent molecules into two-dimensional arrays parallel to the (100) plane.

### Experimental

*N*-Methylformanilide (0.01 mol) and phosphoryl chloride (0.01 mol) were mixed and added into 3-(*p*-tolyl)sydnone (0.01 mol) portion-wise. The reaction mixture was then stirred for about 1 h under cold condition. After standing overnight, it was poured into ice cold water with stirring. The solid obtained was filtered, dried and recrystallized from ethanol. Single crystals suitable for X-ray analysis were obtained from a 1:2 mixture of DMF and ethanol by slow evaporation.

### Refinement

All hydrogen atoms were placed in their calculated positions, with C—H = 0.93 or 0.96 Å, and refined using a riding model with *U*_iso_ = 1.2 or 1.5 *U*_eq_(C). A rotating group model was used for the C10 methyl group.

### Crystal data

**Table:**

C_10_H_8_N_2_O_3_	*F*(000) = 424
*M**_r_* = 204.18	*D*_x_ = 1.448 Mg m^−^^3^
Monoclinic, *P*2_1_/*c*	Mo *K*α radiation, λ = 0.71073 Å
Hall symbol: -P 2ybc	Cell parameters from 6788 reflections
*a* = 10.5663 (4) Å	θ = 2.8–38.9°
*b* = 10.4088 (3) Å	µ = 0.11 mm^−^^1^
*c* = 8.9630 (3) Å	*T* = 100 K
β = 108.222 (1)°	Block, brown
*V* = 936.34 (5) Å^3^	0.71 × 0.30 × 0.19 mm
*Z* = 4	

### Data collection

**Table:**

Bruker APEXII DUO CCD area-detector diffractometer	4906 independent reflections
Radiation source: fine-focus sealed tube	4091 reflections with *I* > 2σ(*I*)
graphite	*R*_int_ = 0.022
φ and ω scans	θ_max_ = 37.5°, θ_min_ = 2.8°
Absorption correction: multi-scan (*SADABS*; Bruker, 2009)	*h* = −18→18
*T*_min_ = 0.926, *T*_max_ = 0.980	*k* = −17→17
14437 measured reflections	*l* = −15→14

### Refinement

**Table:**

Refinement on *F*^2^	Primary atom site location: structure-invariant direct methods
Least-squares matrix: full	Secondary atom site location: difference Fourier map
*R*[*F*^2^ > 2σ(*F*^2^)] = 0.042	Hydrogen site location: inferred from neighbouring sites
*wR*(*F*^2^) = 0.132	H-atom parameters constrained
*S* = 1.07	*w* = 1/[σ^2^(*F*_o_^2^) + (0.0747*P*)^2^ + 0.1421*P*] where *P* = (*F*_o_^2^ + 2*F*_c_^2^)/3
4906 reflections	(Δ/σ)_max_ = 0.001
137 parameters	Δρ_max_ = 0.65 e Å^−^^3^
0 restraints	Δρ_min_ = −0.31 e Å^−^^3^

### Special details

**Table:**

Experimental. The crystal was placed in the cold stream of an Oxford Cryosystems Cobra open-flow nitrogen cryostat (Cosier & Glazer, 1986) operating at 100.0 (1)K.
Geometry. All esds (except the esd in the dihedral angle between two l.s. planes) are estimated using the full covariance matrix. The cell esds are taken into account individually in the estimation of esds in distances, angles and torsion angles; correlations between esds in cell parameters are only used when they are defined by crystal symmetry. An approximate (isotropic) treatment of cell esds is used for estimating esds involving l.s. planes.
Refinement. Refinement of F^2^ against ALL reflections. The weighted R-factor wR and goodness of fit S are based on F^2^, conventional R-factors R are based on F, with F set to zero for negative F^2^. The threshold expression of F^2^ > 2sigma(F^2^) is used only for calculating R-factors(gt) etc. and is not relevant to the choice of reflections for refinement. R-factors based on F^2^ are statistically about twice as large as those based on F, and R- factors based on ALL data will be even larger.

### Fractional atomic coordinates and isotropic or equivalent isotropic displacement parameters (Å^2^)

**Table:**

	*x*	*y*	*z*	*U*_iso_*/*U*_eq_	
O1	0.34006 (6)	0.22147 (5)	0.96639 (6)	0.01912 (11)	
O2	0.26550 (6)	0.39559 (5)	1.06918 (7)	0.02245 (12)	
O3	0.53489 (6)	0.55203 (5)	1.23353 (7)	0.02190 (12)	
N1	0.54958 (6)	0.24689 (5)	1.05402 (6)	0.01524 (10)	
N2	0.45929 (7)	0.17063 (6)	0.96707 (7)	0.01825 (11)	
C1	0.76823 (8)	0.30000 (7)	1.03217 (9)	0.02080 (13)	
H1A	0.7346	0.3773	0.9839	0.025*	
C2	0.90126 (8)	0.26687 (7)	1.05976 (10)	0.02297 (14)	
H2A	0.9571	0.3228	1.0289	0.028*	
C3	0.95243 (8)	0.15115 (7)	1.13297 (9)	0.02082 (13)	
C4	0.86671 (8)	0.06713 (7)	1.17625 (9)	0.02117 (13)	
H4A	0.8996	−0.0106	1.2238	0.025*	
C5	0.73342 (8)	0.09745 (6)	1.14961 (8)	0.01803 (12)	
H5A	0.6768	0.0411	1.1783	0.022*	
C6	0.68736 (7)	0.21424 (6)	1.07890 (7)	0.01561 (11)	
C7	0.35989 (7)	0.33640 (6)	1.05605 (8)	0.01719 (12)	
C8	0.50088 (7)	0.34990 (6)	1.11215 (8)	0.01566 (11)	
C9	0.58136 (8)	0.44999 (6)	1.20780 (8)	0.01744 (12)	
H9A	0.6724	0.4362	1.2517	0.021*	
C10	1.09685 (9)	0.11710 (10)	1.16471 (12)	0.03014 (17)	
H10D	1.1460	0.1928	1.1562	0.045*	
H10A	1.1048	0.0545	1.0895	0.045*	
H10B	1.1319	0.0823	1.2687	0.045*	

### Atomic displacement parameters (Å^2^)

**Table:**

	*U*^11^	*U*^22^	*U*^33^	*U*^12^	*U*^13^	*U*^23^
O1	0.0194 (2)	0.0159 (2)	0.0216 (2)	0.00027 (17)	0.00558 (19)	−0.00320 (16)
O2	0.0212 (3)	0.0188 (2)	0.0285 (3)	0.00347 (18)	0.0094 (2)	−0.00196 (18)
O3	0.0306 (3)	0.0144 (2)	0.0250 (2)	−0.00230 (19)	0.0148 (2)	−0.00363 (17)
N1	0.0194 (3)	0.01228 (19)	0.0155 (2)	−0.00027 (17)	0.00755 (19)	−0.00047 (15)
N2	0.0206 (3)	0.0153 (2)	0.0194 (2)	−0.00021 (19)	0.0070 (2)	−0.00313 (17)
C1	0.0255 (3)	0.0152 (2)	0.0263 (3)	−0.0009 (2)	0.0148 (3)	0.0028 (2)
C2	0.0243 (3)	0.0202 (3)	0.0294 (3)	−0.0039 (2)	0.0157 (3)	−0.0007 (2)
C3	0.0195 (3)	0.0230 (3)	0.0216 (3)	−0.0007 (2)	0.0089 (2)	−0.0034 (2)
C4	0.0220 (3)	0.0192 (3)	0.0237 (3)	0.0025 (2)	0.0091 (3)	0.0025 (2)
C5	0.0207 (3)	0.0143 (2)	0.0211 (3)	0.0000 (2)	0.0095 (2)	0.00184 (19)
C6	0.0186 (3)	0.0133 (2)	0.0172 (2)	−0.00044 (19)	0.0088 (2)	−0.00029 (17)
C7	0.0210 (3)	0.0134 (2)	0.0179 (2)	0.0007 (2)	0.0072 (2)	−0.00046 (18)
C8	0.0195 (3)	0.0127 (2)	0.0166 (2)	0.0000 (2)	0.0083 (2)	−0.00152 (17)
C9	0.0220 (3)	0.0149 (2)	0.0178 (2)	−0.0033 (2)	0.0097 (2)	−0.00218 (19)
C10	0.0195 (3)	0.0387 (4)	0.0328 (4)	0.0012 (3)	0.0091 (3)	−0.0044 (3)

### Geometric parameters (Å, °)

**Table:**

O1—N2	1.3647 (8)	C3—C4	1.3983 (11)
O1—C7	1.4197 (8)	C3—C10	1.5046 (12)
O2—C7	1.2089 (9)	C4—C5	1.3893 (11)
O3—C9	1.2220 (9)	C4—H4A	0.9300
N1—N2	1.2971 (8)	C5—C6	1.3870 (9)
N1—C8	1.3615 (8)	C5—H5A	0.9300
N1—C6	1.4428 (9)	C7—C8	1.4225 (10)
C1—C6	1.3878 (9)	C8—C9	1.4448 (9)
C1—C2	1.3924 (11)	C9—H9A	0.9300
C1—H1A	0.9300	C10—H10D	0.9600
C2—C3	1.3971 (11)	C10—H10A	0.9600
C2—H2A	0.9300	C10—H10B	0.9600
			
N2—O1—C7	110.58 (5)	C4—C5—H5A	121.0
N2—N1—C8	114.64 (6)	C5—C6—C1	122.72 (7)
N2—N1—C6	117.76 (5)	C5—C6—N1	118.03 (6)
C8—N1—C6	127.60 (6)	C1—C6—N1	119.25 (6)
N1—N2—O1	105.66 (5)	O2—C7—O1	120.29 (7)
C6—C1—C2	118.02 (7)	O2—C7—C8	136.05 (6)
C6—C1—H1A	121.0	O1—C7—C8	103.66 (5)
C2—C1—H1A	121.0	N1—C8—C7	105.44 (6)
C1—C2—C3	121.19 (7)	N1—C8—C9	124.90 (6)
C1—C2—H2A	119.4	C7—C8—C9	129.64 (6)
C3—C2—H2A	119.4	O3—C9—C8	122.82 (7)
C2—C3—C4	118.71 (7)	O3—C9—H9A	118.6
C2—C3—C10	120.83 (7)	C8—C9—H9A	118.6
C4—C3—C10	120.45 (7)	C3—C10—H10D	109.5
C5—C4—C3	121.34 (7)	C3—C10—H10A	109.5
C5—C4—H4A	119.3	H10D—C10—H10A	109.5
C3—C4—H4A	119.3	C3—C10—H10B	109.5
C6—C5—C4	118.01 (6)	H10D—C10—H10B	109.5
C6—C5—H5A	121.0	H10A—C10—H10B	109.5
			
C8—N1—N2—O1	−1.23 (7)	N2—N1—C6—C1	120.44 (7)
C6—N1—N2—O1	178.51 (5)	C8—N1—C6—C1	−59.85 (9)
C7—O1—N2—N1	1.09 (7)	N2—O1—C7—O2	179.97 (6)
C6—C1—C2—C3	−0.31 (11)	N2—O1—C7—C8	−0.58 (7)
C1—C2—C3—C4	1.07 (12)	N2—N1—C8—C7	0.88 (7)
C1—C2—C3—C10	−178.99 (8)	C6—N1—C8—C7	−178.84 (6)
C2—C3—C4—C5	−0.81 (11)	N2—N1—C8—C9	−178.07 (6)
C10—C3—C4—C5	179.25 (7)	C6—N1—C8—C9	2.22 (10)
C3—C4—C5—C6	−0.19 (11)	O2—C7—C8—N1	179.18 (8)
C4—C5—C6—C1	1.01 (11)	O1—C7—C8—N1	−0.13 (7)
C4—C5—C6—N1	−178.80 (6)	O2—C7—C8—C9	−1.94 (13)
C2—C1—C6—C5	−0.76 (11)	O1—C7—C8—C9	178.74 (6)
C2—C1—C6—N1	179.05 (6)	N1—C8—C9—O3	166.06 (6)
N2—N1—C6—C5	−59.74 (8)	C7—C8—C9—O3	−12.62 (11)
C8—N1—C6—C5	119.96 (7)		

### Hydrogen-bond geometry (Å, °)

**Table:**

*D*—H···*A*	*D*—H	H···*A*	*D*···*A*	*D*—H···*A*
C1—H1A···O2^i^	0.93	2.41	3.2847 (9)	156
C5—H5A···O3^ii^	0.93	2.60	3.3489 (11)	138

Symmetry codes: (i) −*x*+1, −*y*+1, −*z*+2; (ii) −*x*+1, *y*−1/2, −*z*+5/2.
